# Exendin-4 improves long-term potentiation and neuronal dendritic growth in vivo and in vitro obesity condition

**DOI:** 10.1038/s41598-021-87809-4

**Published:** 2021-04-15

**Authors:** Ming Wang, Gwangho Yoon, Juhyun Song, Jihoon Jo

**Affiliations:** 1 BioMedical Sciences Graduate Program (BMSGP), Chonnam National University, 264 seoyangro, Hwasun, 58128 Republic of Korea; 2 Department of Anatomy, Chonnam National University Medical School, Hwasun, Jeollanam-do 58128 Republic of Korea; 3 NeuroMedical Convergence Lab, Biomedical Research Institute, Chonnam National University Hospital, Jebong-ro, Gwangju, 501-757 Republic of Korea; 4 Department of Neurology, Chonnam National University Medical School, Gwangju, 501-757 Republic of Korea

**Keywords:** Molecular neuroscience, Neuronal physiology, Spine regulation and structure, Synaptic plasticity

## Abstract

Metabolic syndrome, which increases the risk of obesity and type 2 diabetes has emerged as a significant issue worldwide. Recent studies have highlighted the relationship between metabolic imbalance and neurological pathologies such as memory loss. Glucagon-like peptide 1 (GLP-1) secreted from gut L-cells and specific brain nuclei plays multiple roles including regulation of insulin sensitivity, inflammation and synaptic plasticity. Although GLP-1 and GLP-1 receptor agonists appear to have neuroprotective function, the specific mechanism of their action in brain remains unclear. We investigated whether exendin-4, as a GLP-1RA, improves cognitive function and brain insulin resistance in metabolic-imbalanced mice fed a high-fat diet. Considering the result of electrophysiological experiments, exendin-4 inhibits the reduction of long term potentiation (LTP) in high fat diet mouse brain. Further, we identified the neuroprotective effect of exendin-4 in primary cultured hippocampal and cortical neurons in in vitro metabolic imbalanced condition. Our results showed the improvement of IRS-1 phosphorylation, neuronal complexity, and the mature of dendritic spine shape by exendin-4 treatment in metabolic imbalanced in vitro condition. Here, we provides significant evidences on the effect of exendin-4 on synaptic plasticity, long-term potentiation, and neural structure. We suggest that GLP-1 is important to treat neuropathology caused by metabolic syndrome.

## Introduction

A high prevalence of metabolic syndrome which increases the risk of obesity and diabetes, has emerged as a significant global medical issue^1^. A high prevalence of metabolic disorders results in high mortality and morbidity rates as well as increased medical costs^2^. The onset of obesity and diabetes has been related to multiple factors, including an imbalance of energy expenditure, abnormal food intake patterns, environmental changes, chronic stress, and genetic problems^3^.

A recent study has reported that metabolic conditions such as type 2 diabetes and obesity are directly linked to cognitive impairment in the central nervous system (CNS)^4^. In addition, one study has demonstrated that patients with type 2 diabetes and obesity experience accelerated memory loss and impaired verbal fluency compared to normal subjects^5^.

Metabolic imbalance leads to chronic inflammation, insulin resistance, oxidative stress, vascular damage, and impaired lipid and glucose metabolism in the systemic circulation and CNS^6^. These may contribute to changes in brain function and metabolic stress in the body^4^.

Glucagon-like peptide-1 (GLP-1) is a peptide hormone secreted by the gut L-cells. GLP-1 plays a cardinal role in glucose metabolism in specific nuclei of the hindbrain by binding to its specific receptors, called GLP-1 receptors (GLP-1Rs)^7^. GLP-1Rs are widely expressed in various organs such as the pancreas, kidney, bone marrow, lung, gut, and brain^8^.

Endogenous GLP-1 is known to have a short plasma half-life due to fast degradation by dipeptidyl peptidase-4 (DPP-4) in the blood^9^. Therefore, GLP-1R agonists, such as exendin-4, are usually used to treat patients with metabolic complications^10^.

In metabolic imbalance conditions, GLP-1 and GLP-1R agonists, such as exendin-4, exert anti-diabetic and anti-obese effects, including regulation of glucose-dependent insulin homeostasis and regulation of food intake, weight loss, and level of glucagon^11^. A clinical study has proven that administration of GLP-1RA could improve diabetic pathology in patients with type 2 diabetes^12^.

Furthermore, treatment with GLP-1RA could effectively suppress increased glycemic parameters such as hemoglobin A1c (HbA1c) and fasting glucose, reduce body weight, and suppress secretion of inflammatory cytokine^13^. In addition, GLP-1RA could improve the lipid profile and promote hypothalamic connectivity in the CNS and is involved in several brain functions including feeding satiety and stress^14^.

At the cellular level, GLP-1 has multiple functions, including the activation of protein kinase A (PKA) and 3′,5′-cyclic adenosine monophosphate (cAMP) signaling, the cytoplasmic Ca^2+^ pathway^15^, and various mitogen-activated protein kinase (MAPK) pathways^16^.

In the CNS, GLP-1Rs are observed in diverse brain areas, including the hypothalamus. GLP-1 signaling is involved in several brain functions, including feeding satiety and stress response^17^. Moreover, GLP-1Rs expressed in the hippocampal region are related to learning and memory function^18^.

Recently, the role of GLP-1 and GLP-1RA have been highlighted in the CNS since they may protect neurons against oxidative stress and ultimately prevent the progress and onset of neuronal diseases including Alzheimer's disease, Parkinson's disease, and amyotrophic lateral sclerosis through diverse CNS mechanisms^19^. One study has reported that intracerebroventricular administration of GLP-1 resulted in a dramatic improvement of hippocampus-dependent memory function in rodents^20^. Another study has shown that GLP-1 attenuates cognitive deficit in patients with type 2 diabetes^21^. Furthermore, neuritogenesis by GLP-1 treatment enhances long-term potentiation (LTP) and cognitive function^22^.

As previously mentioned, GLP-1 and GLP-1RA have multiple beneficial effects in neuropathology. In this study, we investigated whether exendin-4, acting as GLP-1RA, restores LTP and promotes synaptic plasticity in the brain of mice fed a high-fat diet (HFD). Moreover, our in vitro study examines the mechanisms involved in the effect of exendin-4 in hippocampal neurons in a metabolic imbalance condition. Our findings demonstrate a significant potential of GLP-1, which may improve cognitive decline in HFD mice by attenuating neuroinflammation, enhancing neural structure, and enhancing LTP.

## Results

### Exendin-4 improved neurite complexity of in vitro primary neurons in metabolic imbalanced condition

**Figure 1:**
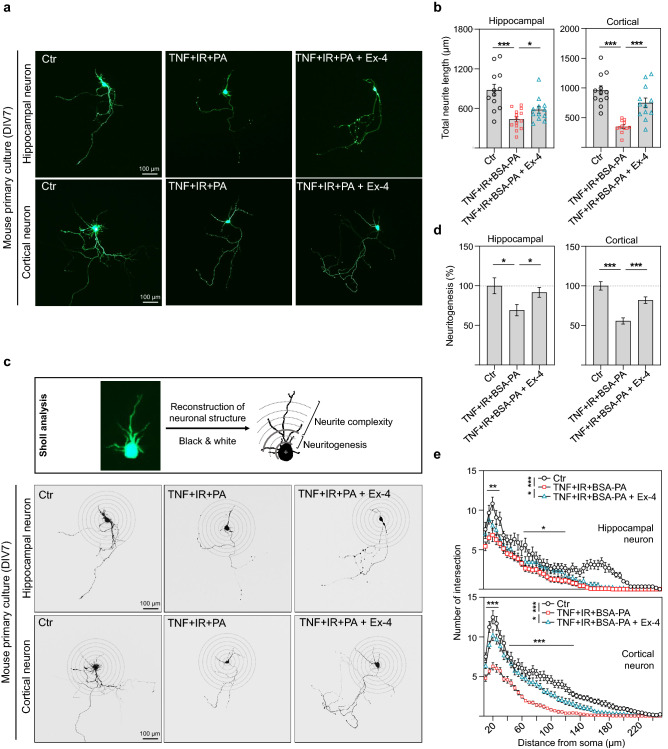
Exendin-4 improved neural complexity of in vitro neurons in metabolic imbalance conditions. (**a**) Representative images (×40) of GFP-positive primary hippocampal and cortical neurons at DIV 7 after treatment with a combination of inducers of metabolic imbalance such as TNF-α, insulin, glucose, palmitate, and a therapeutic agent exendin-4. (**b**) Histogram of total neurite length change after treatment of metabolic imbalance factors and exendin-4 in mouse primary hippocampal and cortical neurons at DIV 7. (**c**) Representative images of reconstructed primary hippocampal and cortical neurons of (**a**). A Sholl analysis was conducted after converting the GFP signal to a black and white reconstruction. The diameter of the first sholl covering the soma was 10 μm, and the radius of the sholl increased by 5 μm. (**d**) Histogram of neuritogenesis change after treatment of metabolic imbalance factors and exendin-4 in mouse primary hippocampal and cortical neurons at DIV 7. (**e**) Histogram of neurite complexity change after treatment of metabolic imbalance factors and exendin-4 in mouse primary hippocampal and cortical neurons at DIV 7. Data information: Ctr.: control, TNF: 25 ng/ml TNF-α, IR: insulin resistance (treatment of 100 nM insulin and 4.5 g/L d-glucose), BSA-PA: 50 μM BSA-conjugated palmitate, Ex-4: 10 nM exendin-4. The length of the scale bar is included in the representative images. Data are expressed as the group mean ± standard error of the mean. In (**b**), (**d**), and (**e**), data are expressed from 12 neurons per independent group in triplicate. Statistical analyses examined the relative significance between each group [(**b**) and (**d**): unpaired two-tail *t*-test with Welch's correction; (**e**) Two-way ANOVA with Bonferroni post-test to compare the replicate means by distance from the soma). ^n.s.^P < 0.05, *P < 0.05, **P < 0.01, and ***P < 0.001.

It has been reported and established that HFD induces neuroinflammation, insulin resistance, and free fatty acid accumulation resulting in cognitive deficits in the brain^23^. Tumor necrosis factor-alpha (TNF-α), a major pro-inflammatory factor, induces insulin resistance through phosphorylation of serine of insulin receptor substrate-1 (IRS-1) in the brain^24^. Palmitate, a saturated free fatty acid, contributes to insulin resistance by suppressing AKT phosphorylation and causes inflammatory responses that are similar to lipopolysaccharide-treated toll-like receptor 4 activation in the brain^25,26^. However, insulin resistance causes inflammation by elevating MCP1 production and accelerates the accumulation of free fatty acid in the brain^27^. These reports imply that HFD causes a complex metabolic imbalanced condition in the brain, suggesting a need for a suitable in vitro model that mimics HFD mice brain. To mimic HFD-induced metabolic-imbalanced neurons, we used a combination method by treating cultured primary hippocampal and cortical neurons with TNF-α (25 ng/ml), insulin (100 mM), and glucose (4.5 g/L) for insulin resistance, and bovine serum albumin (BSA)-conjugated palmitate (50 μM). To examine the effect of exendin-4 on neural structure on metabolic imbalanced condition, we used in vitro models of primary hippocampal and cortical neurons at 7 days in vitro (DIV 7) that have the characteristics such as neuritogenesis and neurite elongation^28^. Cultured metabolic-imbalanced neurons at (DIV 6) were treated with exendin-4 (10 nM) for 24 h. Simultaneously, the treated neurons were transfected with pMAX-GFP for 24 h using a lipid-based transfection method. The representative images of GFP-positive hippocampal and cortical neurons after treatment of TNF-α, insulin resistance, palmitate, and exendin-4 are shown in Fig. 1a. We first analyzed neurite complexity, including the total neurite length and neuritogenesis in metabolic-imbalanced hippocampal and cortical neurons. The total neurite length was significantly shortened in metabolic-imbalanced hippocampal and cortical neurons compared to normal conditioned neurons (Fig. 1a,b). However, exendin-4 increased the neurite length in metabolic-imbalanced hippocampal and cortical neurons, suggesting that exendin-4 promotes neurite outgrowth and recovery of neurite damage by metabolic imbalanced condition. GFP-positive primary neuron was subsequently reconstructed into a black and white 8-bit image to measure the degree of complexity, and a Sholl analysis was performed, as shown in Fig. 1c (upper panel). The percentage of neuritogenesis was calculated from the number of neurites from the soma of primary hippocampal and cortical neurons. Neuritogenesis partially decreased in metabolic-imbalanced neurons compared to the normal condition (Fig. 1c lower panel,d). However, exendin-4 improved neuritogenesis by approximately 10% to 15% in metabolic-imbalanced neurons, suggesting that exendin-4 promotes neurite formation in damaged neurons. While the spread and intersection of neurites from the soma were more simplified in metabolic-imbalanced neurons than in the normal condition, exendin-4 increased the number of intersections of neurites from the soma in metabolic-imbalanced neurons (Fig. 1c lower panel,e). This complexity was particularly evident at a distance of 15 and 25 μm from the soma and between 60 and 120 μm from the soma of hippocampal neurons. In cortical neurons, the complexity was 15 and 30 μm from the soma and 60 and 180 μm from the soma. Taken together, these results suggest that exendin-4 improves neuritic complexity, including neurite length, neuritogenesis, and neuronal network in metabolic-imbalanced neurons, which mimics the HFD mouse brain.

### Exendin-4 improved dendritic spine morphogenesis in in vitro primary neurons in metabolic imbalanced condition

**Figure 2:**
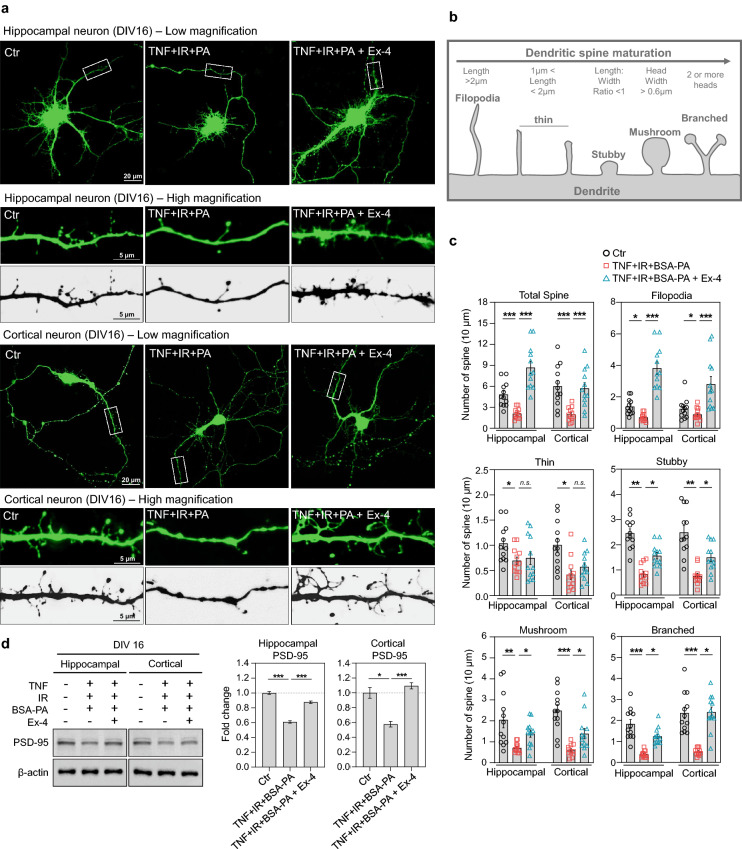
Exendin-4 changes the dendritic spine shape and protein dynamics related to synaptic function in neurons in metabolic imbalance conditions. (**a**) Representative images at low- (×40) and high-magnification (×120) of GFP-positive primary hippocampal and cortical neurons at DIV 16 after treatment of the metabolic imbalance inducing factors such as TNF-α, insulin, glucose, palmitate, and a therapeutic agent exendin-4. The original location of the high-magnification images is indicated by the rectangular box outlined in white in the low magnification images. (**b**) Schematic drawing for the dendritic spine maturation process. Parameters such as the length and width for definition of the spine shape are indicated. (**c**) Histogram for the changes in the shape of the dendritic spine after treatment of metabolic imbalance inducing factors and a therapeutic agent exendin-4 into primary hippocampal and cortical neurons at DIV 16. The number of spine types in the dendrite of 10 μm length was counted to represent the mean. (**d**) The measurement and histogram of the expression of a protein related to synaptic function, PSD-95, after treatment of metabolic imbalance inducing factors and a therapeutic agent exendin-4 into primary hippocampal and cortical neurons at DIV 16. Data information: Ctr.: control, TNF: 25 ng/ml TNF-α, IR: insulin resistance (treatment of 100 nM insulin and 4.5 g/L d-glucose), BSA-PA: 50 μM BSA-conjugated palmitate, Ex-4: 10 nM exendin-4. The length of the scale bar is included in the representative images. Data are expressed as the group mean ± standard error of the mean. In (**a**) and (**c**), data are expressed from 12 neurons per independent group in triplicate. In (**d**), data are expressed from three independent experiments in triplicate. Statistical analyses examined the relative significance between each group (unpaired two-tail *t*-test with Welch's correction). ^*n.s.*^P > 0.05, *P < 0.05, **P < 0.01, and ***P < 0.001.

The dendritic spine number and shape affect synaptic plasticity, memory consolidation, and cognitive deficit in brain diseases^29^. To elucidate whether change in neurite complexity by metabolic imbalance and exendin-4 reflects alteration of dendritic spine morphology that indirectly represent synaptic plasticity and LTP in the mice brain, we examined the effect of exendin-4 on dendritic spine maturation in metabolic-imbalanced primary hippocampal and cortical neurons. We used a combination of TNF-α, insulin, and glucose for insulin resistance, and BSA-palmitate to treat cultured primary hippocampal and cortical neurons. Also, we used in vitro models of primary hippocampal and cortical neurons at (DIV 16) that have the characteristics such as the formation of functional synapses and dendritic spine density^30^. Cultured metabolic-imbalanced neurons at DIV 15 were treated with exendin-4 for 24 h. Simultaneously, the treated neurons were transfected with pMAX-GFP for 24 h using a lipid-based transfection method. Representative images of GFP-positive primary hippocampal and cortical neurons are shown in Fig. 2a. Primary hippocampal and cortical neurons exhibited the shape of the dendritic spine depending on the degree of maturation. The shape of the dendritic spine was classified and measured depending on the length and width (Fig. 2b). We observed a remarkable decrease in the diversity of shape of the dendritic spine in metabolic-imbalanced hippocampal and cortical neurons compared to the normal condition (Fig. 2a,c). The overall shape of the dendritic spine was partially reduced, and the number of stubby, mushroom, and branched dendritic spines markedly decreased in the metabolic-imbalanced hippocampal and cortical neurons compared to the normal condition. However, exendin-4 changed the pattern of spine spread and increased the number of spines in metabolic-imbalanced hippocampal and cortical neurons. In particular, a filopodia-like spine shape was dramatically formed in exendin-4-treated metabolic-imbalanced neurons. Exendin-4 also augmented the number of stubby, mushroom, and branched spines in metabolic-imbalanced neurons, suggesting that exendin-4 increased dendritic spine morphogenesis and maturation after neuronal damage by metabolic imbalance. To examine whether a mature spine-related protein, postsynaptic density-95 (PSD-95), is also affected by metabolic imbalance and exendin-4 treatment, we analyzed PSD-95 expression using western blot analysis. We found that PSD-95 was also remarkably downregulated in metabolic-imbalanced neurons compared to normal condition. However, exendin-4 increased the expression of PSD-95 protein in metabolic-imbalanced neurons (Fig. 2d)**,** suggesting that exendin-4 also increases the synaptic protein expression in metabolic-imbalanced neurons**.** These results indicate that metabolic imbalance reduces the diversity of neural structure and synaptic plasticity and exendin-4 improves dendritic spine dynamics in metabolic-imbalanced neurons and the brain.

### Exendin-4 ameliorated insulin resistance through IRS-1/AKT/GSK-3β and NF-κB pathway in in vitro primary neurons in metabolic imbalanced condition

**Figure 3:**
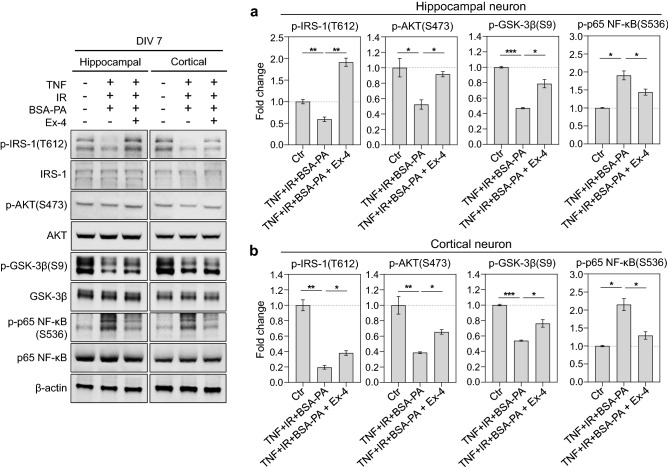
Exendin-4 regulates the phosphorylation of proteins related to insulin signaling and neuroinflammation in in vitro neurons in metabolic imbalance conditions. (**a**) The measurement of phosphorylated and native protein levels after treating with a combination of inducers of metabolic imbalances such as TNF-α, insulin, glucose, palmitate, and a therapeutic agent exendin-4 into primary hippocampal neurons of mice at DIV 7. (**b**) The measurement of phosphorylated and native protein levels after treating with a combination of inducers of metabolic imbalances such as TNF-α, insulin, glucose, palmitate, and a therapeutic agent exendin-4 into mouse primary cortical neurons at DIV 7. Data information: Cont.: control, TNF: 25 ng/ml TNF-α, IR: insulin resistance (treatment of 100 nM insulin and 4.5 g/L d-glucose), BSA-PA: 50 μM BSA-conjugated palmitate, Ex-4: 10 nM exendin-4. Data are expressed as the group mean ± standard error of the mean. Data are expressed from three independent experiments in triplicate. Their native protein normalized a phosphorylated protein, and the corresponding value was expressed as a fold change in the histogram. Statistical analyses examined the relative significance between each group (unpaired two-tail *t*-test with Welch's correction). *P < 0.05, **P < 0.01, and ***P < 0.001.

We evaluated the effect of exendin-4 on metabolic imbalance further. We induced metabolic imbalance using a combination treatment of TNF-α, insulin, and glucose for insulin resistance, and BSA-palmitate into cultured primary hippocampal and cortical neurons. The cultured metabolic-imbalanced neurons at DIV 6 were treated with exendin-4 for 24 h. Protein levels and phosphorylation were analyzed using western blot analysis. An earlier study revealed that HFD induces insulin resistance through dephosphorylation of IRS-1 at tyrosine 612, AKT at serine 473, glycogen-synthase kinase-3beta (GSK-3β) at serine 9 in the brain^31^. In the present metabolic imbalanced model, it induced a similar dephosphorylation of IRS-1, AKT, GSK-3β in primary hippocampal and cortical neurons (Fig. 3a,b), indicating that it mimics the HFD mouse brain well. Exendin-4 increased the phosphorylation of IRS-1, AKT, and GSK-3β in metabolic-imbalanced hippocampal and cortical neurons, indicating that exendin-4 ameliorates insulin resistance in metabolic-imbalanced neurons. Besides, a protein related to HFD-induced neuroinflammation and brain insulin resistance, p65 nuclear factor-κB (NF-κB)^32^, was also highly phosphorylated at serine 536 in metabolic-imbalanced hippocampal and cortical neurons compared to the normal condition (Fig. 3a,b). However, exendin-4 promoted the recovery of the degree of p65 NF-κB phosphorylation in metabolic-imbalanced hippocampal and cortical neurons. These results indicate that exendin-4 improved insulin sensitivity in metabolic-imbalanced hippocampal and cortical neurons.

### Exendin-4 ameliorated insulin resistance and suppressed neuroinflammation in the HFD mouse brain

**Figure 4:**
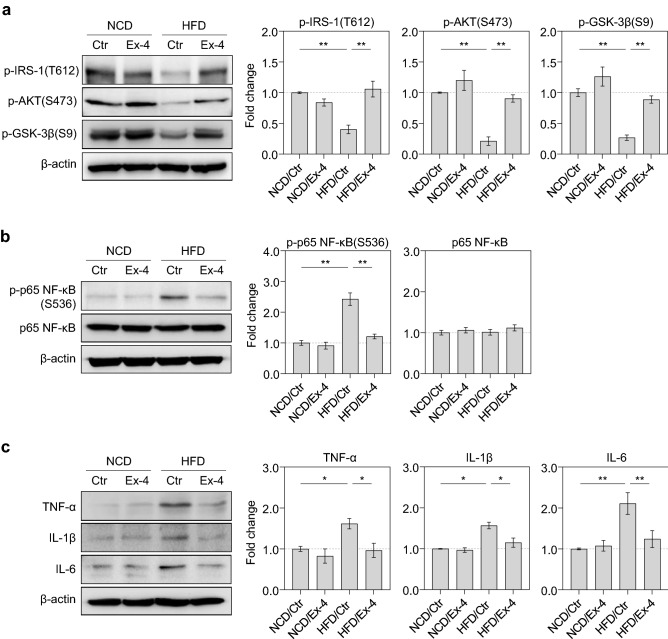
Exendin-4 ameliorated insulin resistance and suppressed neuroinflammation in the HFD mice brain. (**a**) p-IRS-1 (T612), p-AKT (S473), p-GSK-3β (S9) protein levels in the hippocampus between the control and exendin-4 groups in either NCD or HFD-fed mice evaluated by western blot analysis and quantified by densitometric analysis, n = 4, equal to the number of animals. (**b**) p-p65 NF-κB (S536) and p65 NF-κB protein levels in the hippocampus between the control and exendin-4 groups in either the NCD or HFD mice evaluated by western blot analysis and quantified by densitometric analysis, n = 4, equal to the number of animals. (**c**) TNF-α, IL-1β, and IL-6 protein levels in the hippocampus between the control and exendin-4 groups in either the NCD or HFD-fed mice evaluated by western blot analysis and quantified by densitometric analysis, n = 3–4, equal to the number of animals. Data information: Ctr.: control; Ex-4: exendin-4; NCD: normal chow diet; HFD: high-fat diet. Data are expressed as the group mean ± standard error of the mean. Data are expressed from three or four independent experiments in triplicate. Statistical analyses examined the relative significance between each group by ANOVA with *post-hoc* Tukey HSD Test or Games-Howell Test. Differences were considered significant at *P < 0.05, **P < 0.01.

To prove that the effect of exendin-4 on insulin resistance observed in metabolic-imbalanced neurons is similar to that of the hippocampus of HFD mouse brain, the mice were fed a HFD (44.8% fat) for 6 months. The control mice were fed a normal chow diet (NCD) for 6 months. When the NCD and HFD mice reached 6 months of age, exendin-4 (5 μg/g body weight) was intraperitoneally injected once daily for a month. We then evaluated the insulin resistance in the mouse hippocampus by measuring the degree of phosphorylation of IRS-1, AKT, and GSK-3β using western blot analysis. The HFD mice showed a dramatic decrease in phosphorylation of IRS-1, AKT, and GSK-3β in the hippocampus compared to NCD mice (Fig. 4a). The exendin-4 injection significantly increased the phosphorylation of IRS-1, AKT, and GSK-3β in the hippocampus of HFD mice. However, exendin-4 did not change IRS-1, AKT, and GSK-3β phosphorylation in the hippocampus of NCD mice, indicating that exendin-4 improves insulin signaling only in the hippocampus of HFD mice. Together with the observation in metabolic-imbalanced neurons (Fig. 3), our findings reveal that exendin-4 ameliorated insulin resistance through the IRS-1/AKT/GSK-3β pathway in metabolic imbalance in the HFD mouse brain.

HFD potentiated the onset of obesity, microglial activation, and neuroinflammation in the brain by promoting the activation of p65 NF-κB, leading to the secretion of pro-inflammatory cytokines^33^. We measured the p65 NF-κB phosphorylation in the hippocampus of HFD mice using western blot analysis. A significant increase of p65 NF-κB phosphorylation was observed in the hippocampus of HFD mice compared to NCD mice (Fig. 4b). Exendin-4 specifically decreased p65 NF-κB phosphorylation in the hippocampus of HFD mice. However, there was no change in p65 NF-κB phosphorylation with exendin-4 injection in the hippocampus of NCD mice. An assessment of total p65 NF-κB protein levels showed no major difference between vehicle- and exendin-4-injected groups in either NCD or HFD mice. To further explore whether exendin-4 inhibits HFD-induced secretion of pro-inflammatory cytokines, we compared TNF-α, interleukin (IL)-1β, and IL-6 protein levels in the hippocampus of vehicle- and exendin-4-injected groups in either NCD or HFD mice using western blot analysis. We found that exendin-4 injections led to a specific reduction in pro-inflammatory cytokine protein levels in the hippocampus of HFD mice (Fig. 4c). However, exendin-4 did not alter the levels of pro-inflammatory cytokines in the hippocampus of NCD mice, indicating that exendin-4 does not affect the basal level of pro-inflammatory cytokines in the mouse hippocampus. These data indicate that exendin-4 mediates NF-κB signaling and attenuates neuroinflammation by regulating TNF-α, IL-1β, and IL-6 protein levels in the HFD mouse brain.

### Exendin-4 enhanced LTP in the HFD mouse brain

**Figure 5:**
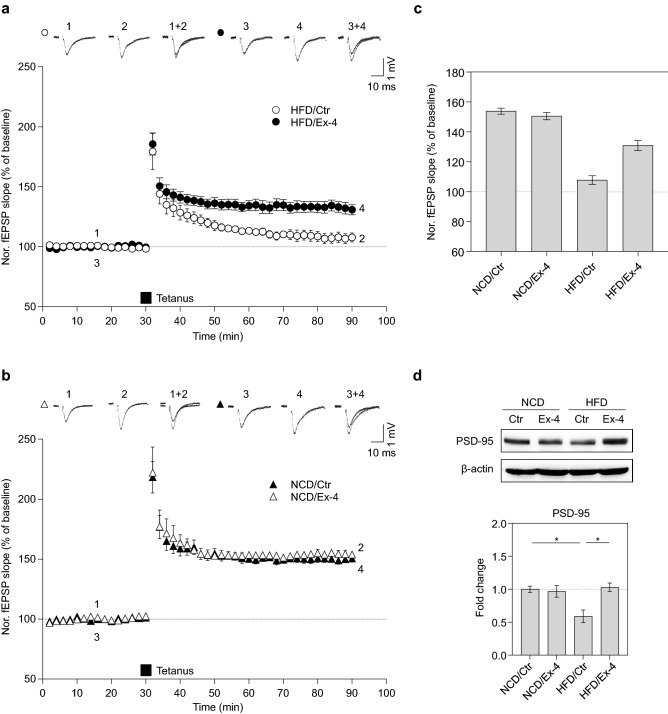
Exendin-4 enhanced LTP in the HFD mice brain. (**a**) HFD-fed wild-type mouse hippocampal LTP was assessed in acute slices of the control group (open circle) and exendin-4 injected group (closed circle), n = 10 per group from 10 animals. (**b**) NCD-fed wild-type mouse hippocampal LTP was assessed in acute slices of the control group (open circle) and exendin-4 injected group (closed circle), n = 10 per group from 10 animals. (**c**) Bar graphs of fEPSP slope after tetanus stimulation between the control and exendin-4 groups in either the NCD or HFD-fed mice hippocampus. (**d**) PSD-95 protein levels in the hippocampus between the control and exendin-4 groups in either NCD or HFD-fed mice evaluated by western blot analysis and quantified by densitometric analysis, n = 4, equal to the number of animals. Data information: Ctr.: control; Ex-4: exendin-4; NCD: normal chow diet; HFD: high-fat diet. All data are expressed as the group mean ± standard error of the mean. Data from western blots are expressed from four independent experiments in triplicate. Statistical analyses examined the relative significance between each group by ANOVA with *post-hoc* Tukey HSD Test. Differences were considered significant at *P < 0.05.

To determine whether the administration of exendin-4 would alleviate the deficit in synaptic plasticity caused by HFD, mice were fed NCD or HFD for 7 months and intraperitoneally injected with either vehicle or exendin-4 daily for a month before being sacrificed. LTP in the hippocampal Schaffer collateral-commissural pathway was evaluated via ex vivo electrophysiological recording in the Cornu Ammonis (CA1) region of vehicle- and exendin-4-treated groups of either NCD or HFD mice. Assessment of LTP showed that exposure to HFD impairs hippocampal synaptic plasticity, with a lower field excitatory postsynaptic potential (fEPSP) slope near the baseline average (107.8 ± 3%, Fig. 5a,c). Notably, exendin-4 injection showed a significant increase in fEPSP slope in HFD mice compared to the vehicle-injected controls (130.9 ± 4%, Fig. 5a,c). Nevertheless, LTP was not facilitated by exendin-4 injection in the NCD mouse hippocampus compared to vehicle-injected controls (control: 154.1 ± 3%, exendin-4: 150.0 ± 1%, Fig. 5b,c), indicating that exendin-4 aids recovery of lower fEPSP in the HFD mouse brain without affecting the basal fEPSP observed in NCD mice. PSD-95 plays a key role in synaptic plasticity. Moreover, HFD is associated with decreased PSD-95 protein levels and dendritic spine density^34^. Therefore, we explored the possible effect of exendin-4 on PSD-95 protein levels in NCD or HFD mice by western blot analysis. We found that exendin-4 specifically upregulated PSD-95 protein levels in the hippocampus of HFD mice (Fig. 5d). However, exendin-4 did not change PSD-95 expression in the hippocampus of NCD mice. These results reveal that exposure to HFD impairs high-frequency stimulation-triggered LTP in the hippocampus. However, the presence of exendin-4 reverses the LTP deficiency caused by HFD, suggesting that exendin-4 improves HFD-impaired hippocampal synaptic plasticity.

Altogether, our results suggest that metabolic imbalance observed in HFD mice exacerbated impaired neuron growth, neuritogenesis, and complexity of neurons in the brain resulting in the depression of LTP and memory consolidation. However, exendin-4 has a therapeutic effect on the changes, leading to an improvement in neural structure and function.

## Discussion

In the present study, we investigated whether GLP-1 contributes to neural structural changes, synaptic plasticity, LTP improvement, neuroinflammation, and insulin sensitivity in the HFD mouse hippocampus and primary cortical neurons in metabolic imbalance conditions.

A previous study has reported that the HFD-induced obese mouse brain showed brain insulin resistance, synaptic failure, impaired neurogenesis, neurotransmitter imbalance, severe neuroinflammation, and memory loss^35^.

Here, we suggest significant and novel findings regarding the therapeutic effects of GLP-1 in hippocampus of the obese mice and metabolic imbalance stress exposure in primary cortical and hippocampal neurons.

Firstly, we observed that exendin-4 helps in the formation of stable neural structure and boosts neurite outgrowth and dendritic spine maturation in hippocampal and cortical neurons in metabolic imbalance conditions. It has been reported that improved memory function affects stable neural connectivity, neurite outgrowth development, and dendritic spine's maturation^36^. One study has demonstrated that dendritic spine morphology and the number of spines in hippocampal region contributes to LTP, learning and memory function, and ultimately to neurological diseases^37^. Dendritic spines form a critical part of excitatory synapses^38^. Therefore, spine morphology and numbers are strongly linked to the strength of synaptic plasticity and memory processes^39^. Dendritic spines change from a thin spine into a mature form and mushroom during the process of spine development^40^. Furthermore, spine morphogenesis and maturation lead to stable neural circuit formation and new spine formation in neurons^41^. During maturation of neural connectivity in neurons, dendritic spines are composed of a large spine head and actin cytoskeleton rich dendrites^42^. A previous study has reported that the expression of synaptic proteins such as PSD-95 and synaptophysin reduces along with synaptic dysfunction in the HFD mouse hippocampus^43^.

Secondly, we observed that exendin-4 attenuates neuroinflammation in HFD brain, hippocampus, and neurons in metabolic imbalance conditions. Metabolic imbalance triggers systemic and central inflammation and induces excessive accumulation of adipose tissue, involving increased secretion of adipokines^44^. Adipose tissue produces many adipokines and pro-inflammatory mediators, including TNF-α, IL-1β, interferon-gamma, and IL-6 in obese people^45^. Moreover, excessive adipose tissue triggers reactive oxygen species production and ultimately leads to chronic inflammation^46^. NF-κB has been considered the central inflammation signaling pathway related to the secretion of inflammatory cytokines^47^. A previous study has reported that an increase in NF-κB activation accelerates accumulation of TNF-α, IL-1β, and IL-6 leading to metabolic diseases such as diabetes^47^. Another study has reported severe inflammation in the hippocampus of diabetic rat brain^48^, resulting in impaired memory function and abnormal emotional behavior^49^. This consequently increases the risk of neurological disorders, such as dementia and stroke^50^. We assume that GLP-1 reduces the expression of pro-inflammatory cytokines and inhibits neuroinflammation by regulating NF-κB signaling.

Next, we found that exendin-4 could ameliorate brain insulin resistance in mice brain and cortical and hippocampal neurons despite metabolic imbalance conditions. Insulin has a neuroprotective effect and promotes energy homeostasis and neuronal differentiation in the CNS^51^. In the diabetic and obese brain, insulin cannot act normally in neuronal cells; this phenomenon has been called "brain insulin resistance," and it leads to neuronal cell death, synaptic failure, and impaired glucose metabolism in the brain^52^. A recent study found that brain insulin resistance can damage hippocampal synaptic plasticity and subsequently aggravate cognitive decline^53^.

Insulin signaling is modulated by serine and threonine phosphorylation of IRS protein^54^. IRS-1 phosphorylation affects downstream signaling, including AKT phosphorylation and GSK-3β activation^55^. In metabolic imbalance conditions such as obesity, IRS-1 dysregulation leads to insulin resistance in the brain and results in neuropathological problems^54^. Several studies have demonstrated that insulin resistance by IRS-1 and IRS-2 dysregulation in the brain causes onset of neurodegenerative disease and results in cognitive decline^54,56^. Serine phosphorylation of IRS-1 elevates amyloid beta accumulation and exacerbates memory deficit in AD brain^57^. In addition, insulin receptor knockout mice showed abnormal synaptic transmission and reduction of AKT signaling activation^58^. It has been reported that exendin-4 induces IRS-1/AKT signaling in the hypothalamus regions^59^. Exendin-4 also promotes the expression of IRS-1 in pancreatic islets^60^. In the present study, exendin-4 accelerated IRS-1/AKT signaling and reduced insulin resistance in the HFD mouse hippocampus and primary cultured cortical and hippocampal neurons. Considering this and the previous findings on impairment of insulin resistance in the obese brain^61^, we hypothesized that GLP-1 improves brain insulin resistance and can ameliorate cognitive decline through the IRS-1/AKT signal pathway in the obese brain.

Finally, we observed that exendin-4 treatment contributes to the improvement of LTP in the HFD mouse hippocampus. LTP has been measured in several brain regions including the hippocampal formation, amygdala, cortex, striatum, and nucleus accumbens^62^. The impairment of LTP in the hippocampus results in poor synaptic plasticity and learning and memory dysfunction, leading to neurodegenerative diseases^63^. A previous study has reported that brain insulin resistance leads to memory loss and inhibits the IRS-1/phosphoinositide 3-kinase/AKT/GSK-3β pathways^64^.

In type 2 diabetes rat brain model, decreased LTP in the hippocampal region has been observed^65^ and linked to insulin signaling defect and inappropriate secretion of neurotransmitters such as GABA^66^. In a different study, GLP-1 receptor knockout mouse showed memory loss in the hippocampus compared to the controls^20^. Another study revealed that GLP-1 administration could rescue memory loss and synaptic dysfunction and decrease GSK-3β activity in the AD mouse model^67^.

GSK-3β activity is an important sign in the brain because GSK-3β regulates synaptic and mitochondrial function and amyloid β toxicity in the brain^68^. Furthermore, reduction of GSK-3β activation results in increase of LTP induction by activation of N-methyl-d-aspartate receptor in the hippocampal CA1 and dentate gyrus regions^69^. Considering our data, we propose that exendin-4 enhances LTP induction in the obese hippocampus and ultimately promotes memory function by reducing GSK-3β activation.

Considering that spine maturation, synaptic plasticity, and neuronal connectivity are reduced in metabolic imbalance conditions such as diabetes and obesity^70^, we suggest that exendin-4 might help recover the neural structure and synaptic plasticity in the obese brain. Therefore, GLP-1 may be vital to the treatment of diverse neuropathology caused by metabolic imbalance.

## Methods

### Animal experiments

Six-week-old male, wild-type C57BL/6J mice were purchased from Koatech (Pyeongtaek, South Korea). They were housed in the Laboratory Animal Research Center, Chonnam National University (CNU), under a 16-h light/8-h dark cycle at 23 °C with 60 ± 10% humidity and given ad libitum access to food and water until 7 months old when the experimental procedures were conducted.

Up to 8 weeks, the mice were fed NCD or HFD (ENVIGO; 44.8% fat, 36.2% carbohydrate, 19% protein), which was continued until they were 7 months old.

The experiments were performed following the recommendations of the "96 Guidance for Animal Experiments", established by the "Animal Ethics Committee" at CNU. The protocol was approved by the "Animal Ethics Committee" at CNU. Study was carried out in compliance with the ARRIVE guidelines.

### Drug treatment

When the mice reached the age of 6 months, Exendin-4 (Abcam, Cambridge, UK) was diluted using sterile saline (vehicle) and administered intraperitoneally once daily for a month (5 μg/g body weight). The mice were subsequently sacrificed for electrophysiological and molecular analysis.

To examine the effects of exendin-4 on neural structural changes in metabolic imbalance conditions, primary hippocampal and cortical neurons of mice were treated with TNF-α (25 ng/ml, Abcam), insulin (100 nM, Sigma Aldrich, Missouri, USA), d-glucose (4.5 g/L, Thermo Fisher Scientific, Massachusetts, USA), BSA-conjugated palmitate (50 μM), and exendin-4 (10 nM).

TNF-α, insulin, and d-glucose solution were diluted using sterilized 1 × PBS, sterilized acidic distilled water by adjusting the pH to 2.0–3.0 with dilute HCl, and growth media for primary neurons, respectively. Palmitate (Sigma) was conjugated with BSA and diluted using absolute ethanol (Thermo Fisher Scientific). The solution was boiled at 40 °C for at least 2 h while vortexing. The palmitate solution was filtrated using syringe filter (0.2 μm; Millipore, Massachusetts, USA) and mixed with 10% BSA solution at a 1:100 ratio.

To analyze the neural structure and protein phosphorylation related to insulin signaling, primary neurons at DIV 2*,* DIV 4, and DIV 6 were treated with TNF-α, insulin, glucose, and BSA-palmitate. Metabolic imbalanced neurons were treated with exendin-4 for 24 h on DIV 6. To analyze the shape of dendritic spine and PSD-95 protein expression, primary neurons were treated with TNF-α, insulin, glucose, and BSA-palmitate on DIV 10, DIV 12, and DIV 14. Metabolic imbalanced neurons were treated with exendin-4 for 24 h on DIV 15.

### Primary neuron culture and transfection

Primary hippocampal and cortical neuron cultures were prepared according to the Animal Care Guidelines of CNU, South Korea.

Primary neuronal cells were removed from the cerebral hippocampi and cortices of embryonic day 14 (E14) and E17 C57BL/6 mice (Koatech). Hippocampal and cortical regions of embryonic mice were incubated in dissection/dissociation medium [1× Hank's balanced salt solution, 1× sodium pyruvate, 1% glucose 100 mM 4-(2-hydroxyethyl)-1-piperazineethanesulfonic acid] for 15 min and triturated using a pipette. The cells were seeded into multi-well plates containing poly-l-lysine coated glass coverslips (Paul Marienfeld, Lauda-Königshofen, Germany). They were allowed to settle in a plating medium (minimum essential medium with Earle's balanced salts, 10% FBS, 0.45% glucose, 1 × sodium pyruvate, 1× GlutaMAX, and 100 U/ml penicillin–streptomycin) for 1 h for attachment. Plating medium was replaced with maintenance medium (Neurobasal medium, 1× B-27 supplement, 1× GlutaMAX, and 100 U/ml penicillin–streptomycin) for growing cells; which was replaced once every 3 days.

For visualization of neuronal structure (neuritic complexity and dendritic spine density), primary neuronal cells at DIV 5 and DIV 14 were transfected with pMAX-GFP plasmid (Lonza, Basal, Switzerland) with FuGENE 6 transfection reagent (Promega, Madison, WI, USA) for 48 h, according to the manufacturer's instructions. After 48 h, cells were fixed using 2% paraformaldehyde (GeneALL, Seoul, South Korea). They were mounted on glass slides using VectaMount solution (VECTOR, Burlingame, USA). The slides were visualized and images captured using an LSM 700 confocal microscope (ZEISS, Oberkochen, Germany) using 40× objective and 10× ocular lens.

### Western blotting with neuronal cells and hippocampus brain tissue

Primary neuronal cells were lysed with ice-cold RIPA buffer (Translab, Daejeon, South Korea) for 15 min. Per manufacturer's instructions, the protein concentration was quantified using a BCA assay kit (Thermo Fisher Scientific). Proteins (20 μg) were electrophoresed on 10% sodium dodecyl sulfate–polyacrylamide gel, which was transferred onto a polyvinylidene difluoride (PVDF) membrane activated by methanol for 10 min. The PVDF membrane was incubated in blocking solution (5% BSA and skimmed milk for phosphorylated and native forms of the protein, respectively) for 1 h 30 min at room temperature, followed by incubation with primary antibodies (1:2000) overnight at 4 ℃. Primary antibodies: p-IRS-1 (Tyr612; Invitrogen, California, USA.), anti-IRS-1 (Invitrogen), p-AKT (Ser473; Cell Signaling, Massachusetts, USA), anti-AKT (Cell Signaling), p-GSK-3β (Ser9; Cell Signaling), anti-GSK-3β (Santa Cruz, Texas, USA), p-p65 NF-κB (Ser536; Cell Signaling), anti-p65 NF-κB (Abcam), PSD-95 (Cell Signaling), and β-actin (Cell Signaling). After incubation with secondary antibodies (1:5000; Santa Cruz) conjugated with horseradish peroxidase for 1 h at room temperature, the membrane was incubated and visualized using ECL solution (Thermo Fisher Scientific) and Fusion Solo software (Vilber, Marne-la-Vallée, France). Protein expression was measured using Fusion Solo and normalized to β-actin and native protein levels.

Hippocampi lysates were prepared in cold RIPA buffer (AKR-190; Cell Biolabs, San Diego, CA, USA) with protease inhibitor cocktail (210205; Cell Biolabs, Inc.). Then, 30–40 μg of proteins were separated on 10–12% SDS–polyacrylamide gel and transferred to PVDF membranes (Millipore, Bedford, MA, USA). The distribution of PSD-95, p-p65 NF-κB (S536), p65 NF-κB, IL-1β, IL-6, TNF-α, p-IRS-1 (T612), p-AKT (S473), p-GSK-3β (S9), or β-actin (Cell Signaling, Danvers, MA, USA) spots were verified using each antibody. The immunoblots were incubated with specific secondary antibodies (Abcam) for 2 h at room temperature, and the bands were detected using the ECL detection system (Millipore, Bedford, MA, USA). We provide the blottings in supplemantary information.

### Slice preparation and electrophysiology experiments

Acute hippocampal slice preparation and electrophysiological recording were performed similar to a previous study^71^. The mice were sacrificed between 9:00 and 10:00 a.m. by cervical dislocation. The brain was placed in cold aCSF containing 124 mM NaCl, 3 mM KCl, 26 mM NaHCO_3_, 1.25 mM NaH_2_PO_4_, 2 mM CaCl_2_, 1 mM MgSO_4_, and 10 mM glucose. With midsagittal cut, the hemispheres were separated and one hemisphere was stored in aCSF until required. Hippocampal slices were isolated and cut transversely (400 μm thick) using a McIIwain tissue chopper (Mickle Laboratory Engineering Co. Ltd., UK) and stabilized for 1 h in aCSF with 95% O_2_/5% CO_2_ of gas at room temperature.

Hippocampal slices were transferred to a recording chamber perfused with oxygenated aCSF (28–29 °C). To record fEPSPs, stimulating bipolar electrodes were placed on the Schaffer collateral pathway. fEPSPs were assessed with glass microelectrodes prepared on a micropipette puller (P-1000; Sutter Instrument, Novato, CA, USA) with 3 M NaCl (3–5 MΩ) inside. After a stable baseline was established for 30 min, LTP was evoked by two tetanus stimulation strains (100 Hz for 1 s with a 30 s interval). fEPSPs were evaluated for at least 1 h. Stimulus intensity during tetanus stimulation was the same as the test pulse. Data were collected using NI USB-6251 data acquisition module (National Instruments, Texas, USA), amplified by Axopatch 700B amplifier (Axon Instruments, CA, USA), and using WinLTP software (http://www.winltp.com).

### Neurite length, neuritogenesis, and neuritic complexity analyses

Images of mouse primary hippocampal and cortical neurons were analyzed using ImageJ to quantify neurite length, neurogenesis, and neuritic complexity. Morphology of selected primary neurons was reconstructed using a manual tracing method. Twelve neurons per group were selected and analyzed in a blinded manner. Using a Sholl analysis plugin in ImageJ, the selected soma center was used to quantify size-related parameters such as the number of intersections and total neuritic length, from radii between 10 and 440 μm, with a 5-μm step size. For neuritogenesis, the number of neurites on soma was sorted from radii between 10–15 μm. Samples per radius were set at 3 and the degree of polynomial fit was selected as "best-fitting degree". Among the Sholl profile list, we selected and combined data in intersection columns^72^.

### Dendritic spine analyses

Over 10 neurites per neuron were considered as a value and 12 values per group were analyzed in a blinded manner. Dendritic spines were classified and measured using ImageJ, according to their shape: filopodia (> 2-μm long with no detectable head), long and short thin (< 2 μm and > 1 μm long with a detectable head < 0.6-μm wide stubby (length: width ratio < 1), mushroom (detectable head > 0.6-μm wide), and branched (≥ 2 heads)^72^.

### Statistical analyses

Statistical analyses were performed using SPSS version 21.0 (IBM Corp., Armonk, NY, USA) and Prism version 8.0 (GraphPad, San Diego, USA). Data are expressed as the mean ± standard error of mean. Data were analyzed using unpaired two-tail *t*-test with Welch's correction, one-way analysis of variance (ANOVA) followed by Bonferroni's post hoc multiple comparisons tests, and two-way ANOVA with Bonferroni post-test to compare replicate means by distance from the soma. P-value < 0.05 was considered statistically significant (Supplementary Information).

## Supplementary Information

Supplementary Information.
