# Supplementary material for: Exendin-4 improves long-term potentiation and neuronal dendritic growth in vivo and in vitro obesity condition
Source: Sci Rep. 2021 Apr 15;11:8326. doi: 10.1038/s41598-021-87809-4 (PMC8050263; doi:10.1038/s41598-021-87809-4)
Supplement: Supplementary file 1 — Supplementary Information. [file 41598_2021_87809_MOESM1_ESM.pdf]

Supplementary Information File

Exendin-4 improves long-term potentiation and neuronal dendritic growth in vivo and in vitro obesity condition

Ming Wang[1+], Gwangho Yoon[2+], Juhyun Song[2*], Jihoon Jo[1,3,4*]

[1]Department of Biomedical Sciences, BK21 PLUS Center for Creative Biomedical Scientists at Chonnam National University, Research Institute of Medical Sciences, Chonnam National University Medical School, Gwangju 501-757, Republic of Korea

[2]Department of Anatomy, Chonnam National University Medical School, Hwasun 58128, Jeollanam-do, Republic of Korea

[3]NeuroMedical Convergence Lab, Biomedical Research Institute, Chonnam National University Hospital, Jebong-ro, Gwangju 501-757, Republic of Korea

[4]Department of Neurology, Chonnam National University Medical School, Gwangju 501-757, Republic of Korea

[+] These authors equally contributed to this study

***Correspondence to:**

Juhyun Song, Ph.D., Department of Anatomy, Chonnam National University Medical School, Hwasun 58128, Jeollanam-do, Republic of Korea, Tel: +82-61-379-2706; E-mail: juhyunsong@chonnam.ac.kr

Jihoon Jo, Ph.D., Department of Biomedical Sciences, Chonnam National University Medical School, 61469, 160, Baekseo-ro, Dong-gu, Gwangju, Republic of Korea, Tel: +82-(0)62-220-4419; E-mail: Jihoon.Jo@jnu.ac.kr

Supplementary Information 1

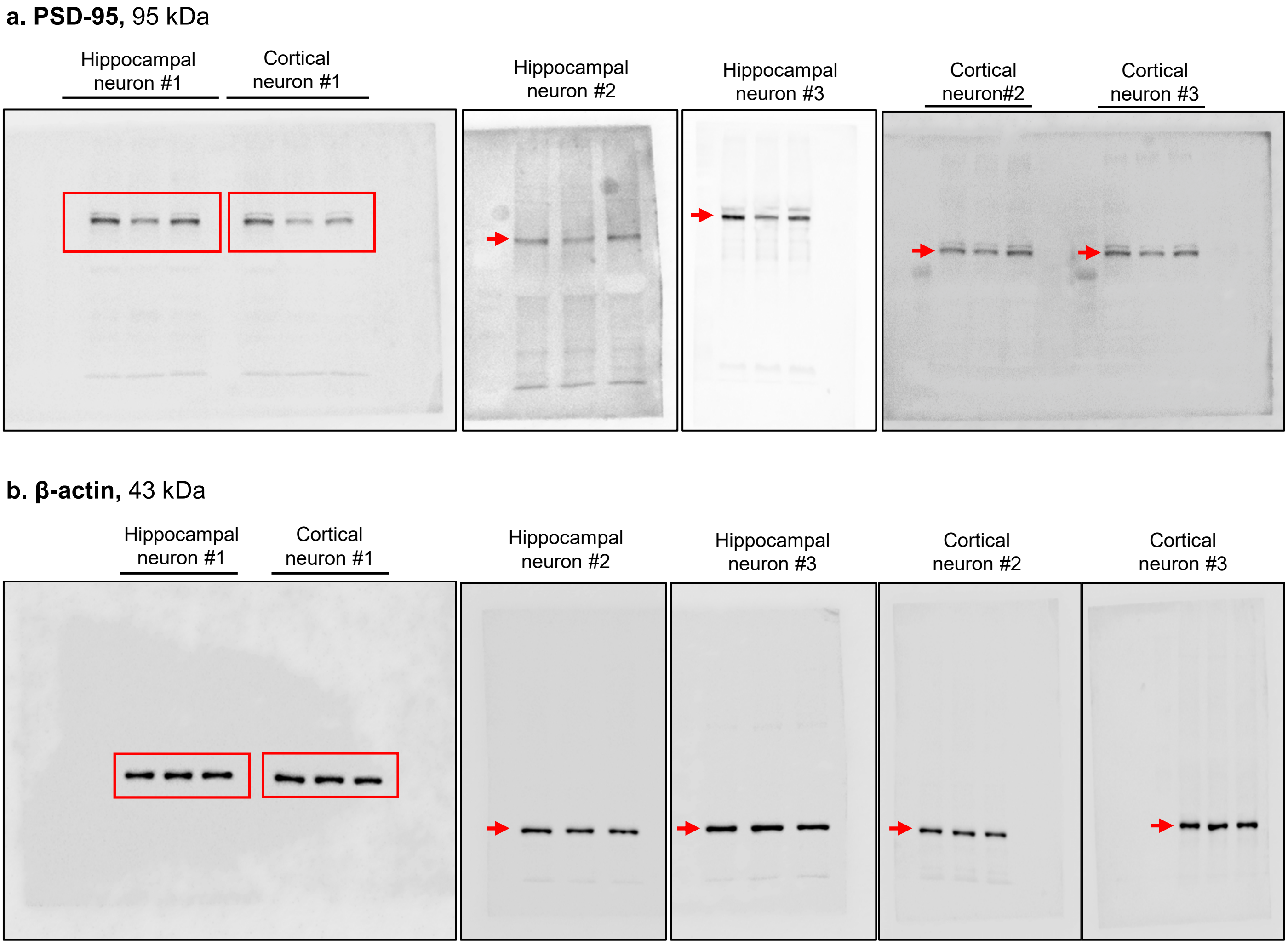

Supplementary information 1. Full-length immunoblots in Figure 2d. **a.** Immunoblot showing changes in PSD-95 protein expression by metabolic imbalance and exendin-4 in mouse primary hippocampal and cortical neuron. **b.** Immunoblot showing β-actin protein expression by Metabolic imbalance and Exendin-4 in mouse primary hippocampal and cortical neuron.

Western blotting was performed by integrating biological triplicates of each group, and the expression of proteins was quantified after technical triplicates. Red box: representative cropped region of immunoblot presented in Figure 2d. Red arrow: the bands observed at the expected size of protein of interest.

Supplementary Information 2

a. p-IRS-1 (T612), approx. 160-185 kDa

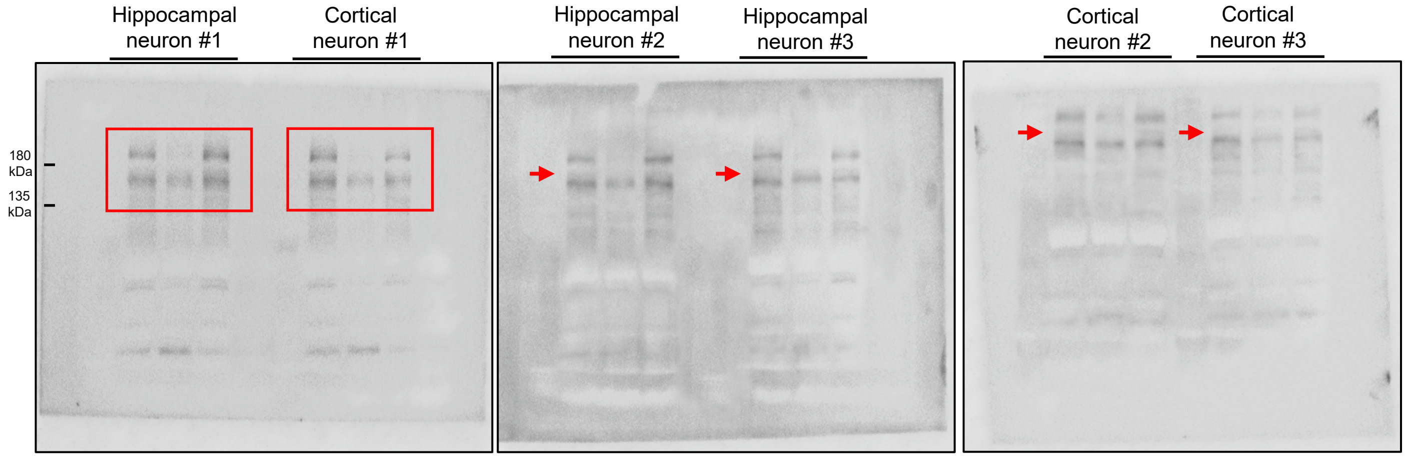

b. IRS-1, approx. 160-185 kDa

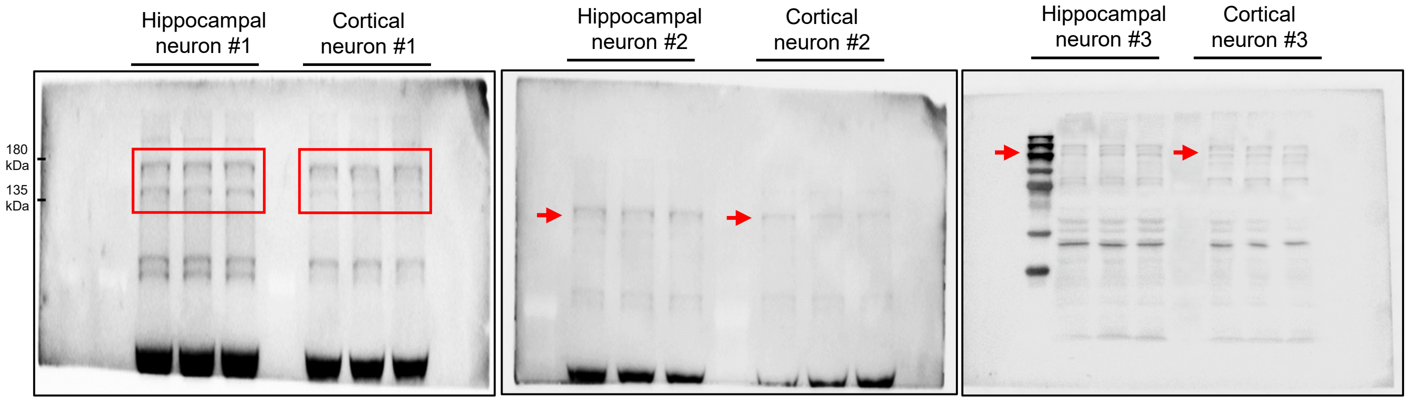

c. p-AKT (S473), 60 kDa

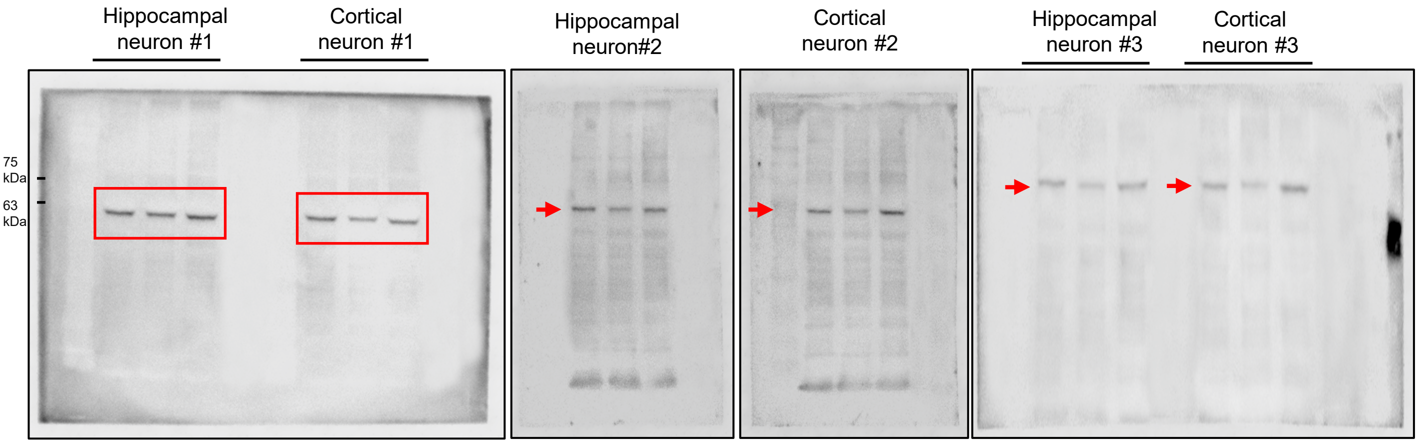

d. AKT, 60 kDa

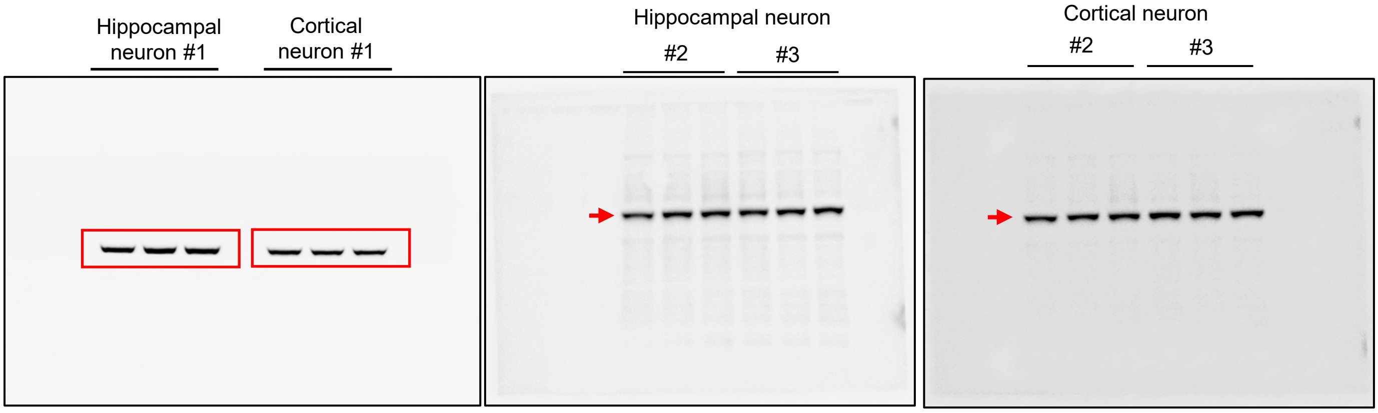

Supplementary Information 2

e. p-GSK-3β (S9), 47 kDa

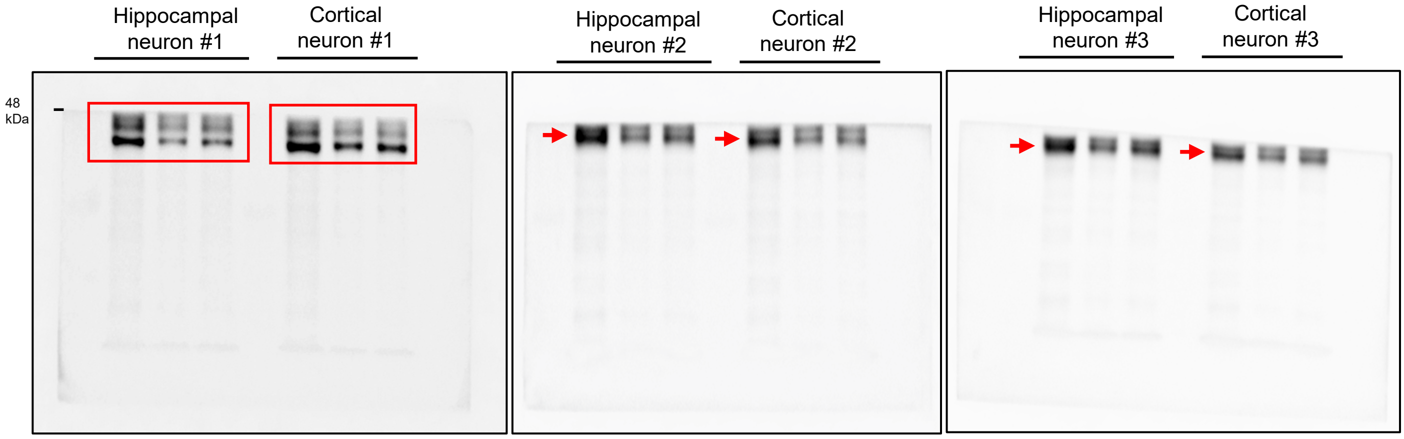

f. GSK-3β, 47 kDa

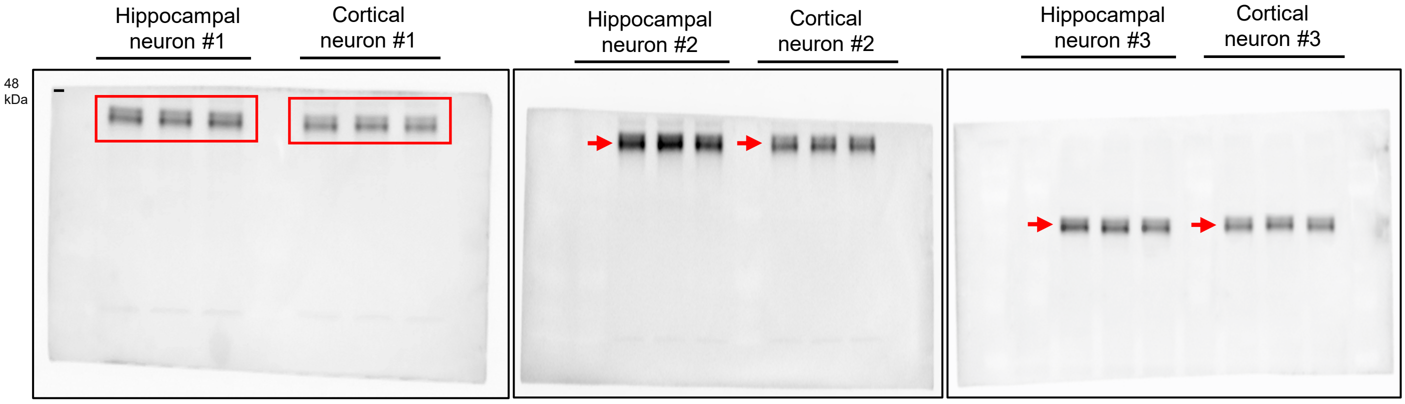

g. p-p65 NF-κB (S536), 65 kDa

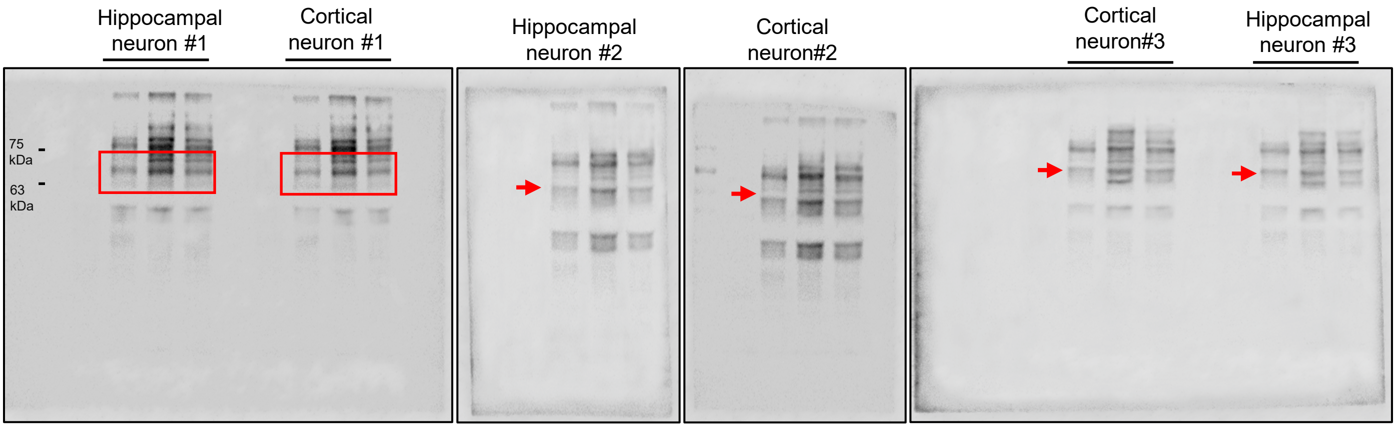

h. p65 NF-κB, 65 kDa

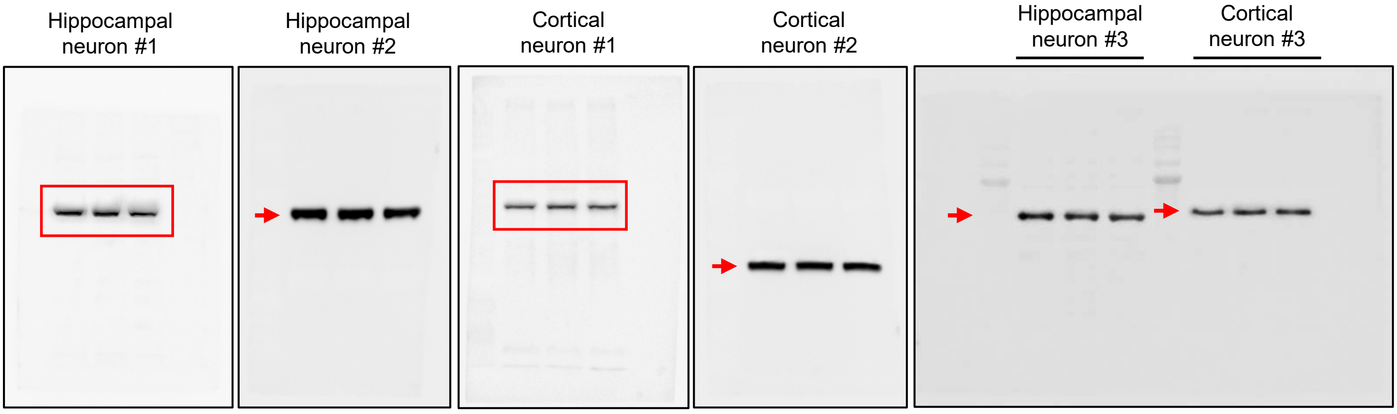

Supplementary Information 2

i. β-actin, 43 kDa

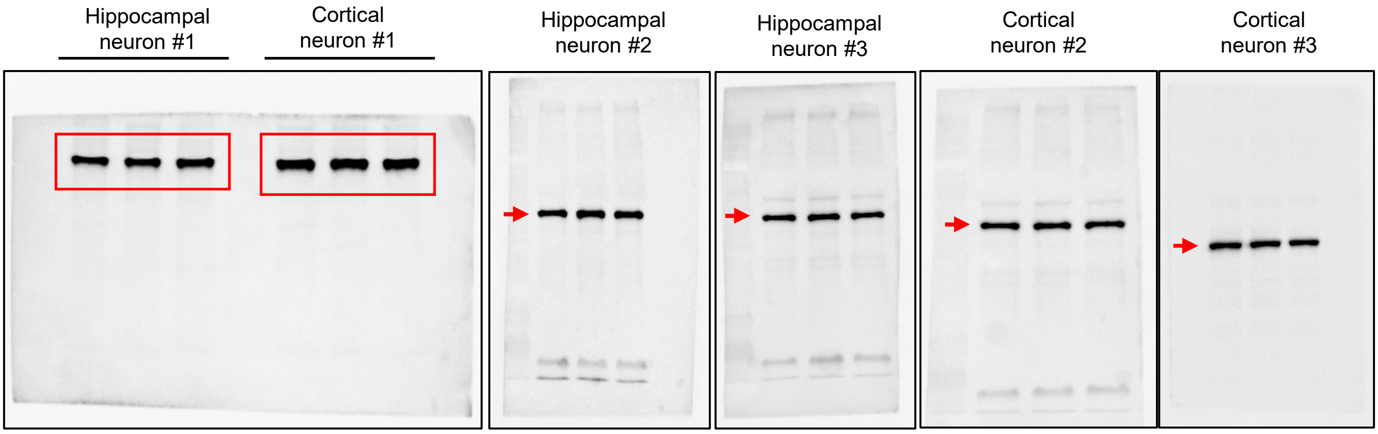

Supplementary information 2. Full-length immunoblots in Figure 3a and 3b. **a.** Immunoblot showing changes in IRS-1 phosphorylation at tyrosine 612 residue by metabolic imbalance and exendin-4 in mouse primary hippocampal and cortical neuron. **b.** Immunoblot showing changes in IRS-1 protein expression by metabolic imbalance and exendin-4 in mouse primary hippocampal and cortical neuron. **c.** Immunoblot showing changes in AKT phosphorylation at serine 473 residue by metabolic imbalance and exendin-4 in mouse primary hippocampal and cortical neuron. **d.** Immunoblot showing changes in AKT protein expression by metabolic imbalance and exendin-4 in mouse primary hippocampal and cortical neuron. **e.** immunoblot showing changes in GSK-3β phosphorylation at serine 9 by metabolic imbalance and exendin-4 in mouse primary hippocampal and cortical neuron. **f.** Immunoblot showing changes in GSK-3β protein expression by metabolic imbalance and exendin-4 in mouse primary hippocampal and cortical neuron. **g.** Immunoblot showing changes in p65 NF-κB phosphorylation at serine 536 residue by metabolic imbalance and exendin-4 in mouse primary hippocampal and cortical neuron. **h.** Immunoblot showing changes in p65 NF-κB protein expression by metabolic imbalance and exendin-4 in mouse primary hippocampal and cortical neuron. **i.** Immunoblot showing changes in β-actin protein expression by metabolic imbalance and exendin-4 in mouse primary hippocampal and cortical neuron.

Western blotting was performed by integrating biological triplicates of each group, and the expression of proteins was quantified after technical triplicates. Red box: representative cropped region of immunoblot presented in Figure 3a and 3b. Red arrow: the bands observed at the expected size of protein of interest.

Supplementary Information 3

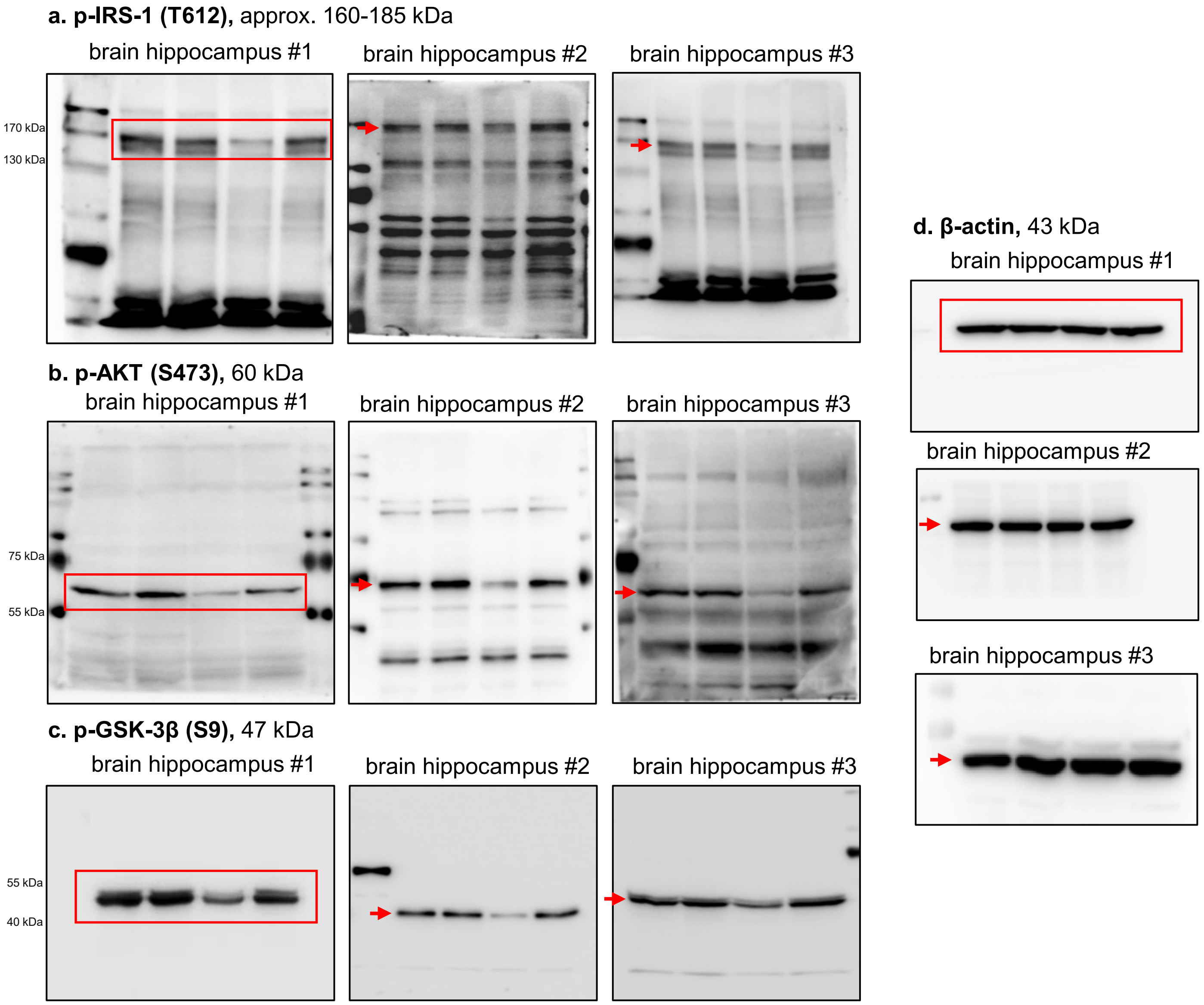

Supplementary information 3. Full-length immunoblots in Figure 4a. **a.** Immunoblot showing changes in IRS-1 phosphorylation at tyrosine 612 residue by high-fat diet and exendin-4 in mouse brain hippocampus. **b.** Immunoblot showing changes in AKT phosphorylation at serine 473 residue by high-fat diet and exendin-4 in mouse brain hippocampus. **c.** Immunoblot showing changes in GSK-3β phosphorylation at serine 9 by high-fat diet and exendin-4 in mouse brain hippocampus. **d.** Immunoblot showing changes in β-actin protein expression by high-fat diet and exendin-4 in mouse brain hippocampus.

Western blotting was performed by integrating biological quadruplicates of each group, and the expression of proteins was quantified after technical triplicates. Red box: representative cropped region of immunoblot presented in Figure 4a. Red arrow: the bands observed at the expected size of protein of interest.

Supplementary Information 4

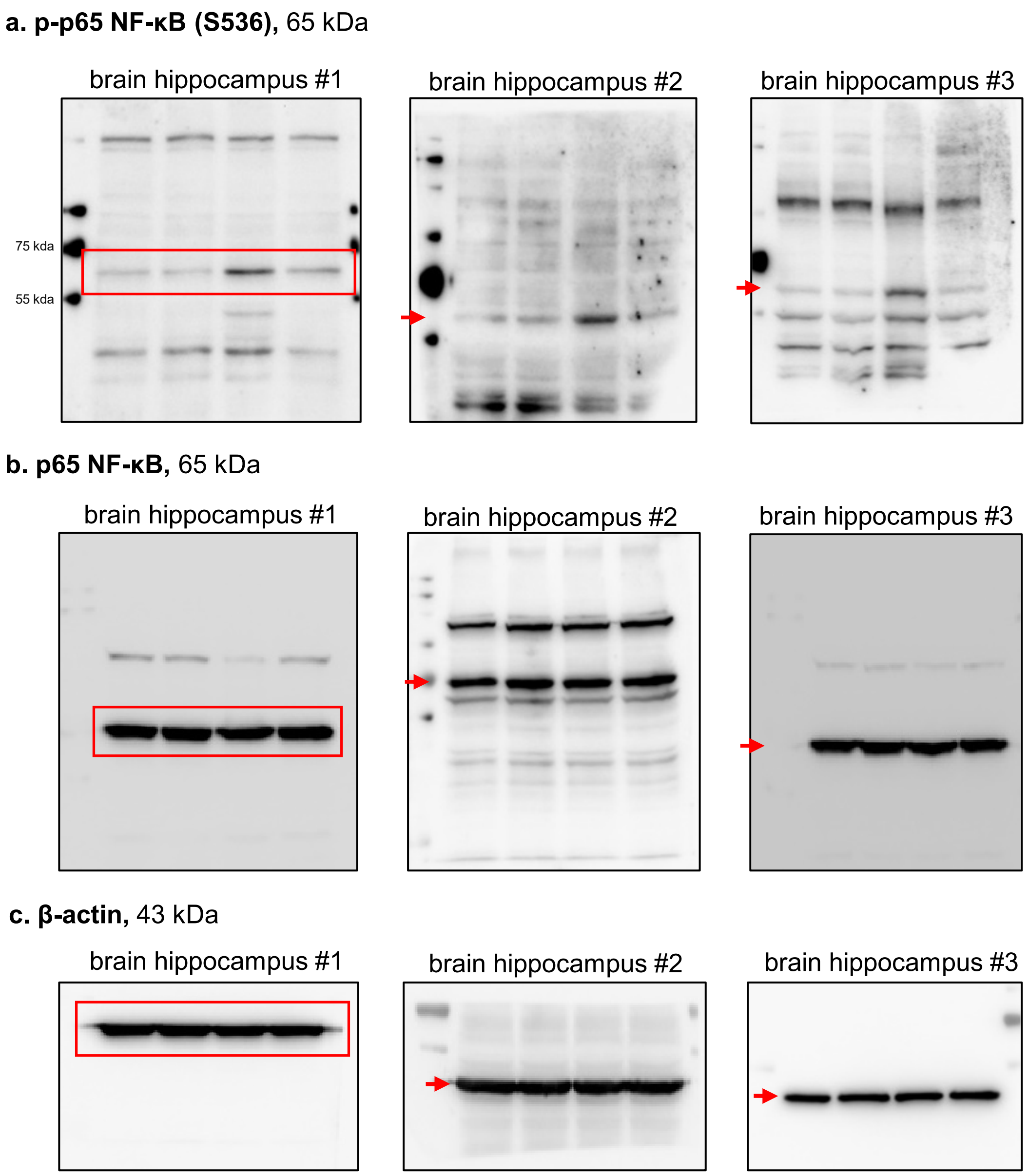

Supplementary information 4. Full-length immunoblots in Figure 4b. **a.** Immunoblot showing changes in p65 NF-κB phosphorylation at serine 536 residue by high-fat diet and exendin-4 in mouse brain hippocampus. **b.** Immunoblot showing changes in p65 NF-κB protein expression by high-fat diet and exendin-4 in mouse brain hippocampus. **c.** Immunoblot showing changes in β-actin protein expression by high-fat diet and exendin-4 in mouse brain hippocampus.

Western blotting was performed by integrating biological quadruplicates of each group, and the expression of proteins was quantified after technical triplicates. Red box: representative cropped region of immunoblot presented in Figure 4b. Red arrow: the bands observed at the expected size of protein of interest.

Supplementary Information 5

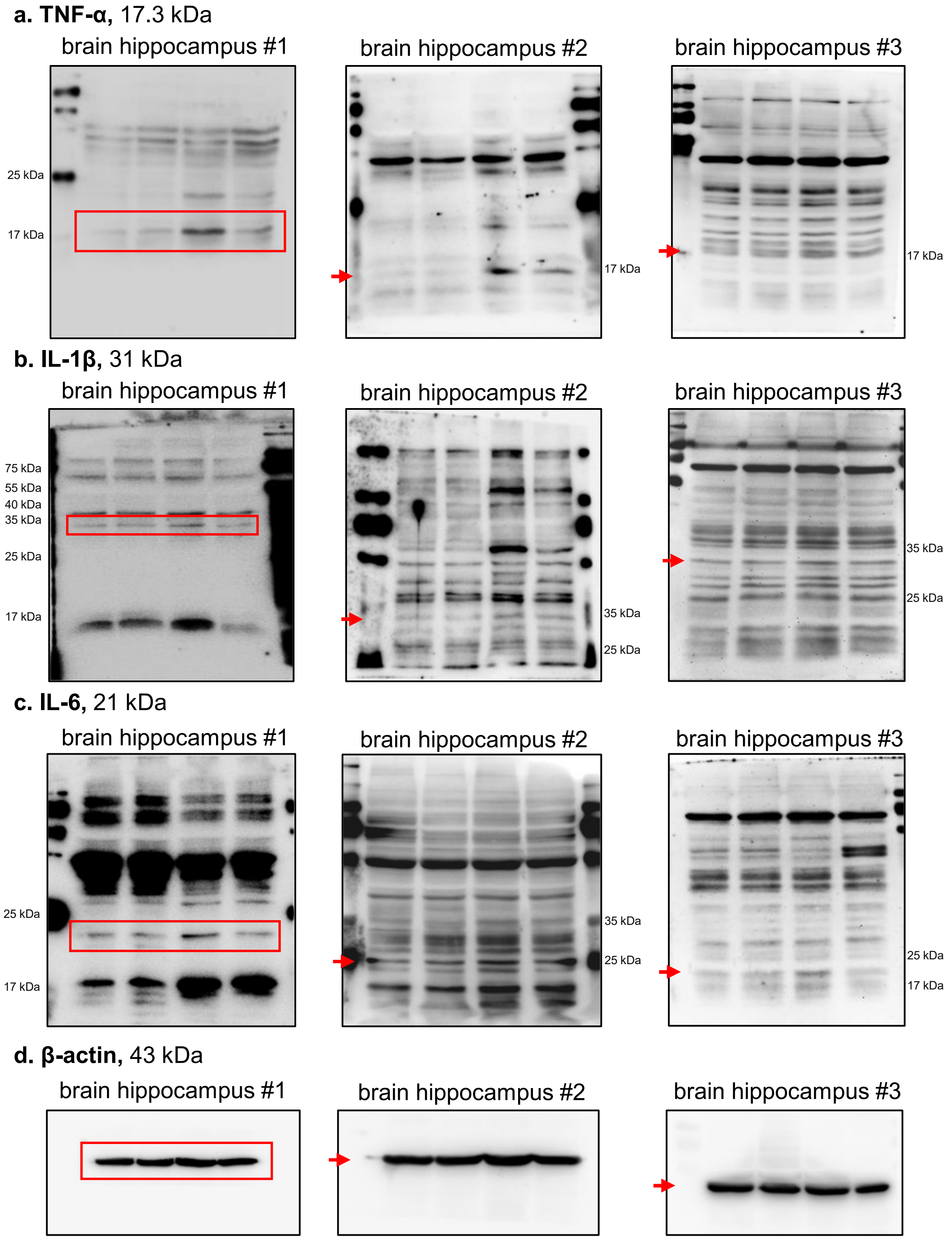

Supplementary information 5. Full-length immunoblots in Figure 4c. **a.** Immunoblot showing changes in TNF-α protein expression by high-fat diet and exendin-4 in mouse brain hippocampus. **b.** Immunoblot showing changes in IL-1β protein expression by high-fat diet and exendin-4 in mouse brain hippocampus. **c.** Immunoblot showing changes IL-6 protein expression by high-fat diet and exendin-4 in mouse brain hippocampus. **d.** Immunoblot showing changes in β-actin protein expression by high-fat diet and exendin-4 in mouse brain hippocampus.

Western blotting was performed by integrating biological quadruplicates of each group, and the expression of proteins was quantified after technical triplicates. Red box: representative cropped region of immunoblot presented in Figure 4c. Red arrow: the bands observed at the expected size of protein of interest.

Supplementary Information 6

a. PSD-95, 95 kDa

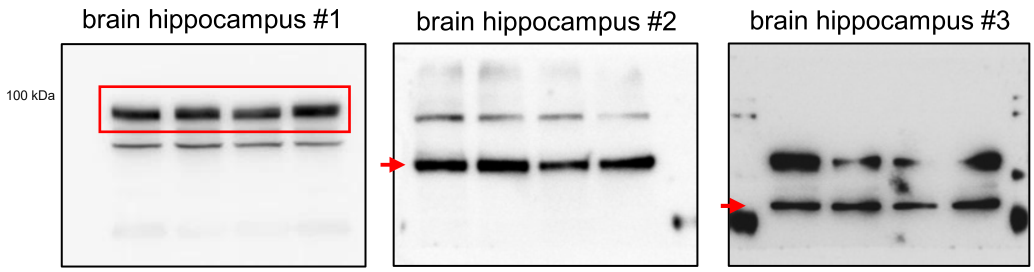

b. β-actin, 43 kDa

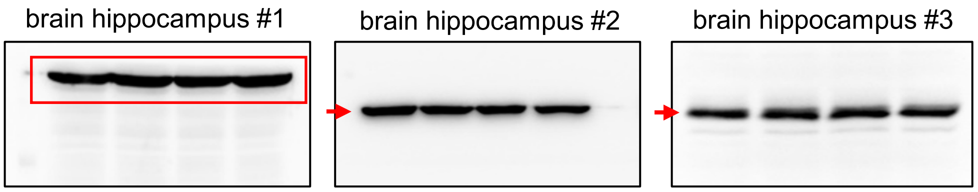

Supplementary information 6. Full-length immunoblots in Figure 5d. **a.** Immunoblot showing changes in PSD-95 protein expression by high-fat diet and exendin-4 in mouse brain hippocampus. **b.** Immunoblot showing changes in β-actin protein expression by high-fat diet and exendin-4 in mouse brain hippocampus.

Western blotting was performed by integrating biological quadruplicates of each group, and the expression of proteins was quantified after technical triplicates. Red box: representative cropped region of immunoblot presented in Figure 5d. Red arrow: the bands observed at the expected size of protein of interest.
